# Organizational identification moderates the effects of perceived COVID‐19 safety climate on COVID‐19 safety behavior in employees' personal life: A social identity approach

**DOI:** 10.1111/aphw.70057

**Published:** 2025-07-03

**Authors:** Philipp Hubert, Sascha Abdel Hadi, Malte Roswag, Andreas Mojzisch, Jan Alexander Häusser

**Affiliations:** ^1^ Department of Psychology Justus‐Liebig‐University Giessen Giessen Germany; ^2^ Institute of Psychology University of Hildesheim Hildesheim Germany; ^3^ Department of Education and Psychology Freie Universität Berlin Berlin Germany

**Keywords:** COVID‐19, health behavior, organizational climate, organizational identification, safety behavior, social identity

## Abstract

Organizations play a pivotal role in fostering employee safety behavior. Building on the social identity approach, we argue that organizational identification facilitates the internalization of organizational norms and values. Thus, organizational identification should strengthen the influence of organizational climate on employee behavior. More specifically, we predicted that the relationship between perceived COVID‐19 safety climate and COVID‐19 safety behavior (both at work and outside of work) would be stronger the more employees identify with their organization. To test this hypothesis, we conducted a two‐wave lagged study with 709 employees after the lifting of government‐imposed COVID‐19 restrictions. Results showed that perceived COVID‐19 safety climate predicted COVID‐19 safety behavior at work, but not in the non‐work domain. Organizational identification moderated the relationship between perceived COVID‐19 safety climate and COVID‐19 safety behavior in the non‐work domain, but not at work. In particular, the positive link between safety climate and safety behavior outside of work emerged only for employees who strongly identified with their organization. Our findings highlight that organizational identification not only influences employee behavior within the workplace but also shapes how employees apply organizational safety norms in their non‐work domain.

## APPROACH

Recently, there has been growing interest in how organizational measures improve employee health and safety behaviors—a trend accelerated by the COVID‐19 pandemic (Huang et al., 2024; Hubert et al., 2022; Roswag et al., 2023, 2024; Sinclair et al., 2020). Safety climate (Zohar & Luria, 2005), which refers to employees' perceptions of safety‐related policies, practices, and procedures, has been proposed as a key factor contributing to employee health and safety (Clarke, 2006; Loh et al., 2019).

However, research on the influence of safety climate remains inconclusive: Meta‐analytic findings suggest that the effect sizes of safety climate on related outcomes tend to be rather small and exhibit high variance across studies (Clarke, 2006). This variability indicates that the relationship between safety climate and employees' safety behavior is contingent upon additional variables that have yet to be integrated into theoretical models of safety climate.

In our research, we argue that the extent to which COVID‐19 safety climate impacts employee behavior critically hinges on employees' organizational identification (Haslam et al., 2009, 2018; Steffens et al., 2021). While safety climate refers to the social context of an organization (Beus et al., 2023), organizational identification reflects the degree to which an individual perceives an overlap between the self and the organization (Haslam, 2012). Hence, organizational identification should be an important factor to boost employees' internalization processes of their perceived organizational context (Häusser et al., 2020).

Moreover, during the COVID‐19 pandemic—and this extends to other infectious diseases (e.g., respiratory diseases)—the effectiveness of organizational safety measures relies on their implementation beyond the workplace. Consider, for example, an employee who strictly followed COVID‐19 safety protocols in the workplace but neglected basic safety measures outside of work. In such cases, the intended protective influence of safety climate may be compromised. We argue that when employees strongly internalize a safety climate, its influence is more likely to extend beyond the workplace and into the non‐work domain. Hence, when organizational identification is high, the spill‐over of organizational climate into the non‐work domain should be more likely.

The major objectives of the present study are two‐fold: First, we examine how perceived safety climate influences safety behavior both in the workplace and beyond. Specifically, within the context of the COVID‐19 pandemic, we examined perceived COVID‐19 safety climate, a focused form of safety climate (Hubert et al., 2022; Roswag et al., 2023, 2024), and COVID‐19 safety behavior after the discontinuation of governmental COVID‐19 prevention measures. As governmental regulation of non‐work behavior declined during this phase of the pandemic, certain health behaviors (e.g., wearing face protection or practicing social distancing) may have remained relevant to individuals' health outcomes (Agyapon‐Ntra & McSharry, 2023). Using a time‐lagged design with two measurement points, we examined the relationship between perceived COVID‐19 safety climate and COVID‐19 preventive behaviors, both at work and in the non‐work domain. We decided to do so because COVID‐19 safety behavior played a pivotal role both within and outside the organization's boundaries.

Second, building on the social identity approach (Haslam, 2012; Tajfel & Turner, 1979), we argue that the relationship between perceived safety climate and health behavior is more pronounced for employees with higher organizational identification. Since organizational identification increases the likelihood of adopting and internalizing organizational norms (Ashforth et al., 2000; Lee et al., 2015; van Dick et al., 2008) we hypothesize that it moderates the relationship between perceived COVID‐19 safety climate and COVID‐19 safety behavior both at work and beyond. Especially, in the case of spill‐over effects of safety climate from the workplace to the non‐work domain, organizational identification may serve as a key boundary condition for the effects of organizational climate (Busse et al., 2017), helping to explain when such spillover occurs. In conclusion, the current research aims to examine whether the effects of safety climate on employee safety behavior vary across work and non‐work settings as a function of organizational identification.

### Perceived COVID‐19 safety climate and health behavior in work and non‐work settings

Organizational climate is a fundamental characteristic of organizations, shaped by how employees interpret and perceive the policies, practices, and procedures they experience, as well as the behaviors they see as expected, supported, and rewarded (Schneider et al., 2013). Therefore, employees' perception of their organization's social environment forms the foundation of their individual representation of organizational climate (Beus et al., 2023). Previous research suggests that when examining the impact of climate on employees' attitudes and behaviors, individual perceptions of climate (referred to as perceived climate) should be considered. In contrast, when the focus is on understanding how a group collectively perceives climate, researchers should rely on aggregated employee perceptions of shared organizational climate (Beus et al., 2023; Kessler, 2019; Loh et al., 2019).

It is also important to note that organizational climate is best considered as a construct that has a referent (Schneider et al., 2013). Thus, a climate is a climate of something, for example, a climate of service (Schneider et al., 1998) or safety (Zohar & Luria, 2005). One of the earliest developed focused climates was safety climate, which highlights the impact of management actions on workplace safety behavior and employee health (Zohar, 1980). Today, there is strong meta‐analytic evidence for the importance of safety climate for employees' health and safety (Alruqi et al., 2018; Clarke, 2006; Griffin & Curcuruto, 2016; Jiang et al., 2019). Building on safety climate research, several focused health climates were developed, and there is mounting evidence for a connection between focused types of safety climate and safety behaviors (e.g., Loh et al., 2019; Roswag et al., 2023).

As a response to the COVID‐19 pandemic, Hubert et al. (2022) introduced perceived COVID‐19 safety climate as a specific type of safety climate, following the framework established by Zohar and Luria (2005). To respond to the circumstances of that time, Hubert et al. (2022) adapted the concept of safety climate to address the specific needs and challenges posed by the pandemic. The concept captures the extent to which the management demonstrates concern for employees' health, particularly in preventing infections during the COVID‐19 pandemic. Hubert et al. found support for the positive impact of COVID‐19 safety climate on adherence to COVID‐19 guidelines, both in the workplace and, notably, beyond. Furthermore, this line of research has revealed its potential to foster other health‐related behaviors, such as employees' willingness to get vaccinated and their actual vaccination uptake (Roswag et al., 2023, 2024).

While these results are in line with previous studies on the effects of safety climate on organizational safety outcomes (Loh et al., 2019), Hubert et al. (2022) provided evidence for the extended influence of safety climate on safety behavior in the non‐work domain. The spill‐over effects from work to non‐work contexts are of particular importance when the target behaviors have implications for non‐work setting (as, for example, in the case of infection prevention). So far, only a few other studies suggest that the consequences of organizational climate extend beyond the workplace and can also impact employees' personal life and their safety behaviors (Naveh & Katz‐Navon, 2015; Rispler & Luria, 2020; Wu et al., 2017). However, the strength of these spill‐over effects varies significantly, indicating that there are boundary conditions that moderate the impact of safety climate on employees' behavior outside the workplace.

In their seminal paper on the connection between work and family life, Edwards and Rothbard (2000) proposed that the transfer of behaviors between work and non‐work domains is most likely to occur when these behaviors have been internalized as habits or scripts and when situational cues in both domains are similar. Thus, the internalization of behaviors and their relevance in employees' personal life seem to be crucial factors affecting the spill‐over process from work to personal life. Given the call to investigate “potential conditions under which climate's influence expands outside the organization” (Naveh & Katz‐Navon, 2015, p. 225) from the outset of climate spill‐over research, we propose that organizational identification could serve as such a condition.

### The social identity approach: organizational identification as a moderator of COVID‐19 safety climate

Organizational identification reflects the degree to which employees perceive themselves as belonging to and defining themselves as part of an organization (Ashforth & Mael, 1989). A useful framework to understand and examine the effects of organizational identification is the social identity approach (Haslam, 2012; Tajfel & Turner, 1979; van Knippenberg et al., 2002), which combines social identity theory (Tajfel & Turner, 1979) and self‐categorization theory (Turner, 1985). This approach suggests that group memberships shape individuals' attitudes, behaviors, self‐concept, and feelings through individuals' identification with a social group or social category (Haslam et al., 2000). Hence, in a work context, an employee's identification as an organizational member is likely to shape how this employee reacts to this specific work environment. Employees with higher organizational identification are more likely to adopt typical organizational behaviors and attitudes as part of their self‐concept (Lee et al., 2015; van Dick et al., 2008). Furthermore, group identification has been found to moderate the effects of perceived norms and values of a group (Tajfel & Turner, 1979; Turner, 1985), making them more influential when group membership is central to one's self‐definition (Duderstadt et al., 2024; Terry & Hogg, 1996).

The social identity approach underpins the relationship between employees and their organization (Kaluza et al., 2020). Therefore, organizational identification likely influences how employees respond to their perception of safety climate and should predict whether employees behave accordingly. In particular, employees with high organizational identification may find a perceived COVID‐19 safety climate more relevant to their self‐concept, enhancing behaviors promoted by this type of climate and therefore adopting and internalizing their organization's attitudes, values, and norms (Ashforth & Schinoff, 2016; Haslam et al., 2000). Therefore, a strong identification would result in a stronger internalization of the norms and values signaled by the climate of the employee's organization (Ashforth et al., 2000). In line with this notion, Kaluza et al. (2020) found that an organizational health climate had a stronger effect on leaders' health mindsets when they identified strongly with their organization. Moreover, it was shown that when employees strongly identify with their leader, an innovative climate amplifies the effect of transformational leadership on employee creativity, thereby acting as a reinforcing factor (Peng Wang & Rode, 2010). Additionally, research on voice climate indicated that highly identified group members were especially likely to share ideas and suggestions (Morrison et al., 2011). In conclusion, there is empirical evidence for a possible moderating effect of organizational identification on the effects of organizational climate. However, these empirical insights are restricted to non‐safety‐related behaviors exhibited in the workplace. Therefore, it is insightful to test whether organizational identification also moderates the effects of safety climate on safety behavior in both work and non‐work contexts.

Building on the social identity approach (Haslam, 2012), we predict that the relationship between perceived COVID‐19 safety climate and COVID‐19 safety behavior is more pronounced the more employees identify with their organization. Hence, we extend previous research on perceived safety climate by investigating organizational identification as a potential moderator for the effects of perceived safety climate. Furthermore, we investigate these moderating effects not only for the relationship between perceived organizational climate and work‐related behavior, but also for behavior outside the workplace. Since highly identified employees integrate behaviors into their self‐concept and internalize organizational norms more deeply (Ashforth et al., 2000; Lee et al., 2015), the relationship between perceived safety climate and non‐work behavior should be stronger among employees with higher levels of identification.

In conclusion, we propose four hypotheses. The first two build on and extend previous research demonstrating a positive relationship between perceived COVID‐19 safety climate and adherence to legally mandated COVID‐19 safety behaviors (Hubert et al., 2022). Our study sought to extend this work by investigating whether COVID‐19 safety climate also predicts voluntary safety behavior, that is, behaviors performed in the absence of legally enforced governmental guidelines.

At the time of data collection (March and April 2022), the COVID‐19 pandemic continued to represent a pervasive, evolving, and highly salient threat that permeated nearly every aspect of employees' lives. The societal and organizational responses to the pandemic were marked by rapidly shifting public health guidelines, organizational policies, and normative expectations. This created an atmosphere of heightened uncertainty and the need for continuous sensemaking (Beus et al., 2023; Schneider et al., 2013). Within this dynamic and disruptive context, we had the unique opportunity to observe how employees developed and acted upon their perceptions of organizational safety climate, not only in formal workplace environments but also in more informal non‐work settings.

Additionally, given the high levels of fatigue regarding COVID‐19 safety measures (Agyapon‐Ntra & McSharry, 2023), it is particularly relevant to examine the effects of perceived safety climate in this context. Moreover, extending previous theory on safety climate, Hypotheses 3 and 4 propose that the relationship between perceived COVID‐19 safety climate and COVID‐19 preventive behavior is moderated by organizational identification, both at work and outside of work. Hence, we hypothesize:

H1: Perceived COVID‐19 safety climate (T1) is positively related to COVID‐19 safety behavior at the workplace (T2).
H2: Perceived COVID‐19 safety climate (T1) is positively related to COVID‐19 safety behavior outside work (T2).
H3: Organizational identification (T1) moderates the relationship between perceived COVID‐19 safety climate and COVID‐19 safety behavior at the workplace (T2), with a stronger relationship for more strongly identified employees.
H4: Organizational identification (T1) moderates the relationship between perceived COVID‐19 safety climate and COVID‐19 safety behavior outside work (T2), with a stronger relationship for more strongly identified employees.

## METHODS

### Sample and procedure

We recruited our sample through Prolific, targeting participants from the USA, UK, and Germany, for the sake of generalizability, as these countries were lifting COVID‐19 measures at the time of the study while being in different phases. Participants needed to work at least 11 hours per week for an organization (excluding self‐employment). Initially, 882 participants joined at T1, but our final sample included only those who participated at both T1 and T2, totaling *N* = 709 participants. No other exclusion criteria were applied. We determined the sample size for our moderation analysis a priori, without implementing any data collection stopping rule, and no analyses were conducted prior to completing data collection and reaching the target sample size. Given the inherent challenges of conducting formal a priori test power analyses for complex path models involving moderators, we based our sample size on general recommendations for studies employing similar designs (Cohen, 2013; Wolf et al., 2013). Attrition t‐tests showed that participants who dropped out from T1 to T2 tended to be younger (*M*_agedropout_ = 32.65, *SD*_agedropout_ = 9.47) compared to our final sample, including all participants that provided data at T1 and T2 (*M*_age_ = 36.88, *SD*_age_ = 11.28), *t*(303) = 5.06, *p* < .001) but there were no differences for any other variables (sex, country, and type of work) across the times of measurement. The final sample included 323 females (45.94%), 380 males (54.05%), and six non‐binary (0.01%) participants. Participants worked on average *M* = 36.75 hours per week (*SD* = 9.09). Of all participants, 484 (68.27%) worked in white‐collar jobs, 115 (16.22%) worked primarily with people (e.g., care‐work), 87 (12.27%) worked mainly in service or in direct customer contact, and 23 (3.24%) worked in the production or shipping of goods. Two hundred fifty‐four participants were from the USA (35.83%), 246 from the United Kingdom (34.70%), and 209 participants were from Germany (29.48%).

T1 data collection took place between March 22 and April 1, 2022, a period when COVID‐19 restrictions had recently been lifted in the UK and the US, and were on the verge of being lifted in Germany. Summarizing the dynamic of the pandemic at that time, the United Kingdom had already transitioned to a “Living with COVID‐19” strategy early in 2022, lifting most legal restrictions and focusing on personal responsibility and vaccination (gov.uk, 2022). By contrast, Germany followed a more cautious approach, gradually easing restrictions from April 2022 while retaining certain mandates like mask‐wearing in specific settings and retaining the option to reintroduce stricter controls if necessary (Bundesministerium für Gesundheit, 2022). The United States had adopted a relatively consistent approach throughout the pandemic, relying on softer public health measures, with a strong focus on vaccination and testing mandates (U.S. Department of Defense, 2022).

Given the rapidly changing nature of COVID‐19 guidelines during this period, we chose a relatively short retest interval. T2 data collection took place between June 27 and July 18, 2022, by which point most COVID‐19 restrictions had been lifted across all three countries. At T1, we measured perceived COVID‐19 safety climate, while COVID‐19 safety behavior and organizational identification were assessed at both times. Participants were informed about the voluntary nature of the study and were assured of confidentiality, anonymity, and the option to discontinue at any time. They received an average compensation of GBP 1.7 per measurement point.

### Measures

#### Perceived organizational COVID‐19 safety climate

We used a seven‐item measure to assess perceived COVID‐19 safety climate (e.g., “My organization offered support and provided me with equipment to deal with the circumstances resulting from the corona pandemic.”; *α* = .91), adapted from existing safety climate scales (Zohar & Luria, 2005) for COVID‐19 (Hubert et al., 2022). Participants rated items on a six‐point scale (1 = absolutely not true, 6 = absolutely true) based on their experiences over the past two years.

#### Organizational identification

Organizational identification was measured using an established four‐item scale (Doosje et al., 1995) including items like “I identify with my organization.,” on a seven‐point scale (1 = strongly disagree, 7 = strongly agree; *α* = .96).

#### COVID‐19 safety behavior at the workplace and in personal life

We assessed COVID‐19 safety behavior using a five‐point scale (1 = absolutely not true, 5 = absolutely true) for workplace (8 items, *α* = .68; e.g., “If I am present at my workplace, I make sure to air it regularly”) and non‐work settings (11 items, *α* = .89; e.g., “I avoid larger groups of people”) by adapting items by Hubert et al. (2022). We complemented this scale by adding new items regarding wearing face masks, using corona self‐tests, or social events that were missing in their initial scale (due to non‐availability at that time) but were relevant for this stage of the COVID‐19 pandemic.

All materials, questionnaires, data, and scripts are available as an OSF project (https://osf.io/fw9kg/?view_only=d16adaa2ff39480d94870b19323de8d8). All the items were translated into German by our research group.

### Data analysis

We conducted statistical analyses using R (4.2.1) (R Core Team, 2022). Hypotheses were tested with path analysis using the lavaan (0.6.12) package and maximum likelihood estimation (Rosseel, 2012). Further analyses used the semTools (0.5.6) package (Jorgensen et al., 2022). To estimate the interaction between perceived safety climate and organizational identification, we used double‐mean centering (Lin et al., 2010).

## RESULTS

**FIGURE 1:**
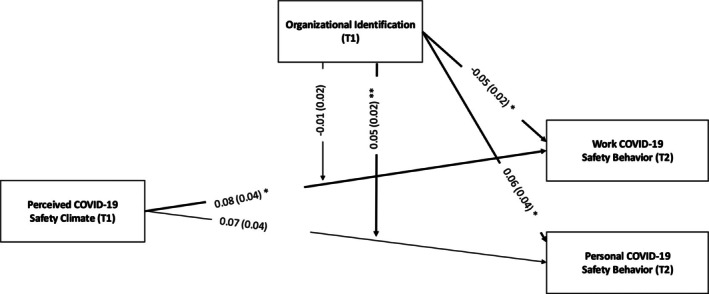
Results of our path analysis, including our moderator analysis. *Note*: The moderator variable was double‐mean centered. * indicates *p* < .05. ** indicates *p* < .01.

Means, standard deviations, and zero‐order correlations of all study variables are shown in Table 1. To confirm that perceived COVID‐19 safety climate and organizational identification are distinct factors, we used a confirmatory factor analysis in which all items of the two scales loaded onto separate factors. We found support for the distinctiveness of both factors: *χ* ^2^(43) = 181.761, *p* < .001, *CFI* = 0.992, TLI = 0.990, RMSEA = 0.029, 90% CI [0.024, 0.033], SRMR = 0.030. Our findings show that perceived COVID‐19 safety climate at T1 predicted workplace COVID‐19 safety behavior at T2 (*b* = .08, *se* = 0.04, *p* = .02, 95% CI [0.01, 0.15]), supporting Hypothesis 1. However, it did not predict safety behavior outside work at T2 (*b* = .07, *se* = 0.04, *p* = .12, 95% CI [−0.02, 0.16]); thus, Hypothesis 2 was not supported. Organizational identification at T1 did not moderate the relationship between perceived COVID‐19 safety climate at T1 and COVID‐19 workplace safety behavior at T2 (*b* = −.01, *se* = 0.02, *p* = .68, 95% CI [−0.03, 0.03]), rejecting Hypothesis 3. However, it did significantly moderate the relationship between perceived COVID‐19 safety climate and non‐work COVID‐19 safety behavior (*b* = .05, *se* = 0.02, *p* < .01, 95% CI [0.02, 0.09]), supporting Hypothesis 4. Model results are displayed in Figure 1 and Table 2.

**TABLE 1:** Means, standard deviations, and correlations.

Variable	*M*	*SD*	1	2	3	4	5
1. Perceived COVID‐19 safety climate	4.61	1.08					
2. Organizational identification at T1	4.84	1.62	.59**				
3. Personal COVID‐19 safety behavior T1	3.40	0.96	.14**	.15**			
4. Personal COVID‐19 safety behavior T2	3.01	1.01	.09*	.10**	.83**		
5. Work COVID‐19 safety behavior T1	3.55	0.72	.09*	−.06	.65**	.57**	
6. Work COVID‐19 safety behavior T2	3.42	0.80	.05	−.04	.65**	.69**	.76**

*Note*: *M* = mean, *SD* = standard deviation.

* *p* < .05.

** *p* < .01.

**TABLE 2:** Results of regression analysis in lavaan.

Variable	Predictor	*b*	*se*	*z*	*p* value	CI lower	CI upper
Personal COVID‐19 safety behavior at T2	Perceived COVID‐19 safety climate	.07	0.04	1.58	.11	−0.02	0.16
	Organizational identification at T1	.06	0.03	2.01	.04	0.03	0.12
	Climate * organizational identification	.05	0.02	2.71	.01	0.02	0.09
Personal COVID‐19 safety behavior at T1	Perceived COVID‐19 safety climate	.10	0.04	2.40	.02	0.02	0.18
	Organizational identification at T1	.07	0.03	2.63	.01	0.02	0.13
	Climate * Organizational identification	.05	0.02	2.85	.01	0.02	0.09
Work COVID‐19 safety behavior at T2	Perceived COVID‐19 safety climate	.08	0.04	2.36	.02	0.01	0.15
	Organizational identification at T1	−.05	0.02	−2.32	.02	−0.10	−0.08
	Climate * Organizational identification	−.01	0.02	−0.58	.95	−0.03	0.03
Work COVID‐19 safety behavior at T1	Perceived COVID‐19 safety climate	.13	0.03	4.04	.00	0.07	0.19
	Organizational identification at T1	−.08	0.02	−3.67	.00	−0.12	−0.04
	Climate * Organizational identification	.01	0.02	0.36	.72	−0.02	0.03

*Note*: The moderator variable was double‐mean centered.

**FIGURE 2:**
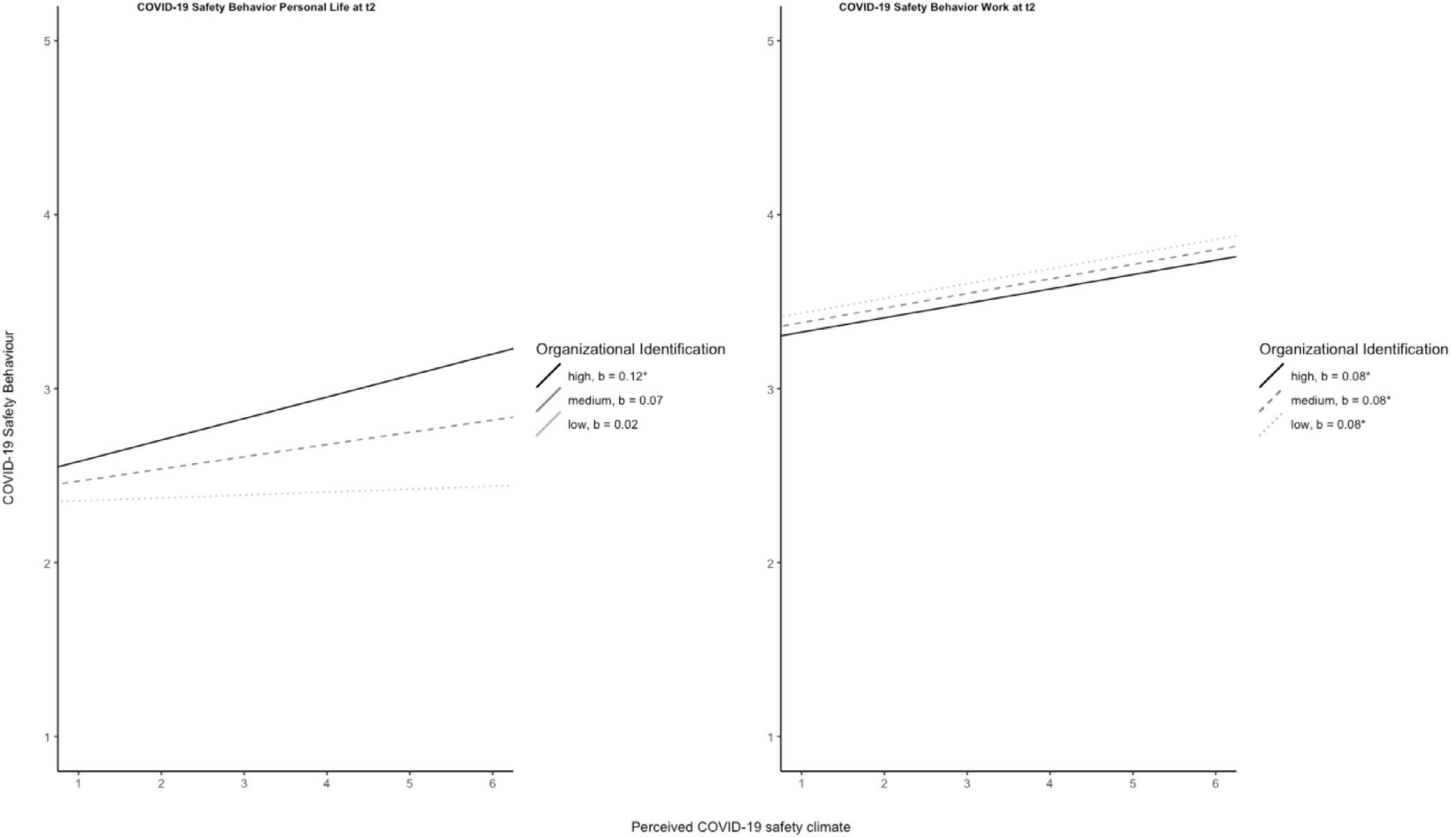
Results of simple slope analysis depicting the effect of different levels of organizational identification on the relationship between perceived COVID‐19 safety climate and COVID‐19 safety behavior. *Note*: The moderator variable was double‐mean centered.

A simple slope analysis for low‐ (−1SD), medium‐ (mean), and high‐identified (+1SD) employees revealed that the positive relationship between perceived COVID‐19 safety climate and non‐work COVID‐19 safety behavior was significant *only* for highly identified employees (*b* = .12, *p* = .02), but not for low‐identified (*b* = .02, *p* = .70) or moderately identified (*b* = .07, *p* = .11) employees. Results are displayed in Figure 2.

## DISCUSSION

In this study, we applied the social identity approach (Haslam, 2012) to examine how perceived COVID‐19 safety climate and organizational identification interact to influence health‐related behavior both within and beyond the workplace. We predicted that stronger organizational identification would enhance the influence of perceived COVID‐19 safety climate on employees' COVID‐19 safety behavior.

To examine this hypothesis, we analyzed data collected after most official COVID‐19 restrictions had been lifted, although expert‐recommended safety guidelines remained in place. We found that perceived COVID‐19 safety climate was positively related to COVID‐19 safety behavior at work, regardless of employees' level of organizational identification. By contrast, perceived COVID‐19 safety climate influenced safety behavior outside of work only among employees who strongly identified with their organization. Thus, organizational identification moderated the relationship between perceived COVID‐19 safety climate and non‐work safety behavior, but not workplace safety behavior.

### Theoretical and practical implications

Employees who perceived a stronger COVID‐19 safety climate at T1 were more likely to engage in workplace safety measures at T2, confirming previous findings (Hubert et al., 2022). However, this relationship was not moderated by employees' level of organizational identification. Albeit speculatively, this pattern may reflect the high salience of organizational norms during the waning of the pandemic, with many organizational measures still in play (e.g., video conferencing, provision of disinfectants), resulting in a relatively standardized and closely monitored safety practices at work. As such, organizational safety behavior may have shown limited variability and been perceived as normative or obligatory, thereby reducing the potential moderating role of organizational identification. Supporting this interpretation, our data revealed lower variance and a smaller decline in workplace safety behavior across time points compared to safety behavior in the non‐work domain (see Table 1).

In contrast, the relationship between COVID‐19 safety climate at T1 and safety behavior in the non‐work domain was moderated by organizational identification. Specifically, perceived safety climate predicted non‐work safety behavior only among employees who strongly identified with their organization. As expected, high organizational identification appeared to facilitate the spillover of organizational norms into individuals' non‐work domains. A strong sense of organizational identification may diminish the psychological boundary between work and non‐work contexts, thereby increasing the likelihood that organizational values are internalized and expressed beyond the workplace (Ashforth et al., 2000).

Another lens through which to interpret these findings is the distinction between compliance and discretionary safety behavior. Workplace safety actions, such as distancing and hygiene, may have been perceived as mandatory, either due to formal regulations or implicit expectations, and thus less reliant on individual motivation or identification. By contrast, safety behaviors in non‐work settings were typically unmonitored and voluntary, more akin to safety citizenship behaviors, that is, discretionary, prosocial acts motivated by personal or collective concern (Curcuruto et al., 2019; Hofmann et al., 2003). In this context, strong organizational identification may have served as a key motivational driver, encouraging employees to enact organizational values even when such behavior was not externally enforced (Lee et al., 2015).

In summary, our findings in the non‐work domain align with the social identity approach to organizational behavior (Haslam, 2012; Haslam et al., 2020; van Knippenberg et al., 2002). When employees strongly identify with their organization, they are more likely to align their personal behavior with organizational norms and values. Although research on the spillover of organizational health climate to employees' personal life is still emerging (Hubert et al., 2022; Naveh & Katz‐Navon, 2015; Roswag et al., 2023; Sonnentag & Pundt, 2016), our data highlight the value of the social identity approach in explaining how safety and health climate effects extend beyond the workplace. Consequently, our study contributes to bridging the gap between safety climate and social identity research and responds to calls for a deeper and more nuanced understanding of the boundary conditions of safety climate (Busse et al., 2017; Schneider et al., 2013). Our findings suggest that even in a context marked by waning governmental oversight and growing fatigue toward COVID‐19 safety measures (Agyapon‐Ntra & McSharry, 2023), perceived safety climate still effectively promoted safety behaviors outside of work—provided employees identified strongly with their organization.

From a practical perspective, it is essential to consider the broader social context in which a safety climate is established. Particularly in situations where other forms of social compliance are lacking, encouraging employees' identification with their organization can enhance the internalization of norms and values, thus serving as a vehicle to influence behavior in other domains of life. Hence, if organizations seek to create a more sustainable impact of safety climate on their employees' health behavior, they should focus on strengthening employees' identification. This can be achieved by fostering positive interactions with leaders (Steffens et al., 2014) and promoting a strong organizational image (Dutton et al., 1994).

### Limitations and directions for future research

One significant limitation of the present study is the absence of an objective measure of COVID‐19 safety behavior. Considering that people often tend to overestimate their own adherence to safety behaviors during a pandemic (Mojzisch et al., 2022), incorporating more objective measures could strengthen the validity of our findings. However, collecting such data, especially in individuals' personal lives, poses significant methodological challenges. We believe that any self‐report bias likely reflects a general better‐than‐average effect, which may introduce random error but not systematically distort the observed relationships.

Moreover, we focused on perceived climate, referring to individual perceptions of organizational climate (James et al., 1978), rather than aggregated perceptions across employees. Kessler (2019) argued that individual perceptions are particularly informative, as they are more likely to trigger immediate psychological and behavioral responses, whereas aggregated climate reflects a more complex interplay of interindividual and organizational dynamics. Supporting this interpretation, a review by Loh et al. (2019) found that shared and individual levels of health‐related climate might differ in their functions, with individual levels of climate being more strongly related to individuals' motivation and behavior and shared climate being more strongly related to group‐level outcomes. However, for broader generalizability, future research should also measure climate at the shared group level.

Another limitation concerns the exclusive focus on the organizational level for both perceived COVID‐19 safety climate and organizational identification. Previous research suggests that the group level of both safety climate (Zohar & Luria, 2005) and (team) identification (Häusser et al., 2020) also plays an important role in shaping safety‐related perceptions and behaviors. Future research should investigate the group level of both perceived safety climate and identification to identify potential processes that emerge within this group level context.

While our findings emphasize the moderating role of organizational identification in extending safety climate effects into the non‐work domain, it is unlikely to be the sole driver of COVID‐19 safety behavior outside the workplace. For example, Auton and Sturman (2022) found that individuals' knowledge of public health restrictions was the strongest predictor of behavioral intentions, surpassing dispositional and demographic factors. This highlights the importance of cognitive clarity and informational access, particularly in contexts where formal enforcement is limited. Future research should therefore examine how organizational climate interacts with individuals' knowledge, beliefs, and broader socio‐political contexts in shaping discretionary health behaviors.

Another area for future research is the conceptual distinction between organizational climate and organizational culture. Although often used interchangeably, these constructs represent related yet distinct phenomena. Organizational climate reflects employees' shared perceptions of enacted policies, procedures, and behaviors, which are visible, communicated, and reinforced in the day‐to‐day work environment (Beus et al., 2023; Schneider et al., 2017). In contrast, organizational culture, represents deeper, enduring values and assumptions (Schneider et al., 2013, 2017). According to recent theoretical work (Beus et al., 2023) climate and culture both emerge from common sensemaking processes but differ in terms of visibility and stability. Given our focus on observable, policy‐driven organizational responses to COVID‐19, organizational climate was the more appropriate construct. Unlike culture, climate can be reliably measured through perceptual survey data and is closely linked to behavioral outcomes in dynamic contexts (Loh et al., 2019). Although we focused on climate, future research may benefit from examining also how organizational culture aligns with identity‐based processes. For example, Tear and Reader (2023) propose that culture may act as a form of “meta‐identity,” offering a more stable foundation for social identity activation. Thus, while our findings highlight how identity moderates climate‐behavior relationships, especially in rapidly changing contexts, future studies could explore whether organizational identification also interacts with organizational culture in shaping long‐term safety behavior, especially in more stable or highly normative environments.

A limitation of the present study is the lack of data on participants' specific work arrangements (i.e., whether they were working on‐site, remotely, or in a hybrid format). During the pandemic, remote work was widely encouraged or mandated as a protective strategy. Although all participants reported working at least 11 hours per week, it is likely—especially given the timing of data collection—that many were still working partially from home, even after restrictions were lifted. This could have blurred the boundary between work and non‐work contexts, potentially attenuating the distinction between the two domains in our data. A clearer separation might have revealed even stronger moderation effects for organizational identification. Nevertheless, research conducted both before and during the pandemic suggests that psychological and behavioral differences between remote and on‐site employees are generally modest (Rudolph et al., 2021). Accordingly, we do not expect this issue to have substantially biased our findings. Still, future research should explicitly assess work location and role salience to better account for evolving workplace configurations in post‐pandemic contexts.

Lastly, an important limitation of our study lies in the volatile and evolving nature of the COVID‐19 pandemic. In particular, public perceptions of COVID‐19 measures likely shifted over the course of data collection, potentially influencing how participants responded at different time points. Therefore, our findings and methods (particularly the time lag between measurement waves) may not be fully generalizable to more stable, non‐pandemic settings. Therefore, future research should aim to replicate our results across a range of settings, examining different facets of safety climate and various forms of employee behavior, such as pro‐environmental climate (Hicklenton et al., 2019) or psychosocial safety climate (Dollard & Karasek, 2010), to better understand the broader applicability of these mechanisms.

## CONCLUSION

This study reveals how perceived safety climate and organizational identification jointly shape employees' safety behavior both in the work and non‐work domain. While perceived safety climate was associated with safety behavior at work regardless of organizational identification, its impact on behavior in the non‐work domain depended on employees' level of identification with their organization. These findings underscore the power of applying a social identity approach to safety climate research (Haslam et al., 2000; van Knippenberg et al., 2002), highlighting organizational identity as a key mechanism through which organizational norms can extend into employees' private lives. To maximize the long‐term effectiveness of safety initiatives, organizations should cultivate a strong sense of identification among employees, thereby ensuring that safety values are internalized.

## CONFLICT OF INTEREST STATEMENT

None of the authors have a conflict of interest to disclose.

## ETHICS STATEMENT

This study was approved by the ethics committee of the University of Hildesheim (Fachbereich 01, Approval Number: 166).

## Data Availability

The data that support the findings of this study are openly available in OSF at https://osf.io/fw9kg/?view_only=d16adaa2ff39480d94870b19323de8d8.
